# A guide to genome mining and genetic manipulation of biosynthetic gene clusters in *Streptomyces*

**DOI:** 10.71150/jm.2409026

**Published:** 2025-04-29

**Authors:** Heonjun Jeong, YeonU Choe, Jiyoon Nam, Yeon Hee Ban

**Affiliations:** 1 Department of Intergrative Molecular and Biomedical Science, College of Biomedical Science, Kangwon National University, Chuncheon 24341, Republic of Korea; 2 Department of Molecular Bioscience, College of Biomedical Science, Kangwon National University, Chuncheon 24341, Republic of Korea; 3 Multidimensional Genomics Research Center, Kangwon National University, Chuncheon 24341, Republic of Korea

**Keywords:** *Streptomyces*, secondary metabolites, biosynthetic gene clusters, genome mining, genetic manipulation

## Abstract

*Streptomyces* are a crucial source of bioactive secondary metabolites with significant clinical applications. Recent studies of bacterial and metagenome-assembled genomes have revealed that *Streptomyces* harbors a substantial number of uncharacterized silent secondary metabolite biosynthetic gene clusters (BGCs). These BGCs represent a vast diversity of biosynthetic pathways for natural product synthesis, indicating significant untapped potential for discovering new metabolites. To exploit this potential, genome mining using comprehensive strategies that leverage extensive genomic databases can be conducted. By linking BGCs to their encoded products and integrating genetic manipulation techniques, researchers can greatly enhance the identification of new secondary metabolites with therapeutic relevance. In this context, we present a step-by-step guide for using the antiSMASH pipeline to identify secondary metabolite-coding BGCs within the complete genome of a novel *Streptomyces* strain. This protocol also outlines gene manipulation methods that can be applied to *Streptomyces* to activate cryptic clusters of interest and validate the functions of biosynthetic genes. By following these guidelines, researchers can pave the way for discovering and characterizing valuable natural products.

## Overview

Natural microbial products, including antibiotics, anticancer agents, and immunosuppressants, are bioactive molecules with a significant potential for drug development. The continuous discovery of various compounds with remarkable structural and biological diversity has underscored the need to explore new biologically active substances (Atanasov et al., 2021; Carroll et al., 2022). Over the past decades, the discovery of natural products has primarily relied on bioactivity-guided isolation, with actinomycetes playing a major role as sources of numerous secondary metabolites with potential pharmacological activities (Demain, 2014). However, by the late 20th century, the discovery process encountered several obstacles. The frequent rediscovery of known compounds and the limitations of traditional low-throughput screening techniques have significantly diminished the likelihood of identifying novel bioactive compounds. This has made the search for novel drug candidates increasingly challenging (Atanasov et al., 2021; Katz & Baltz, 2016). Innovative approaches are required to enhance screening methods and explore microbial diversity for natural product discovery to overcome this bottleneck.

**Fig. 1.**
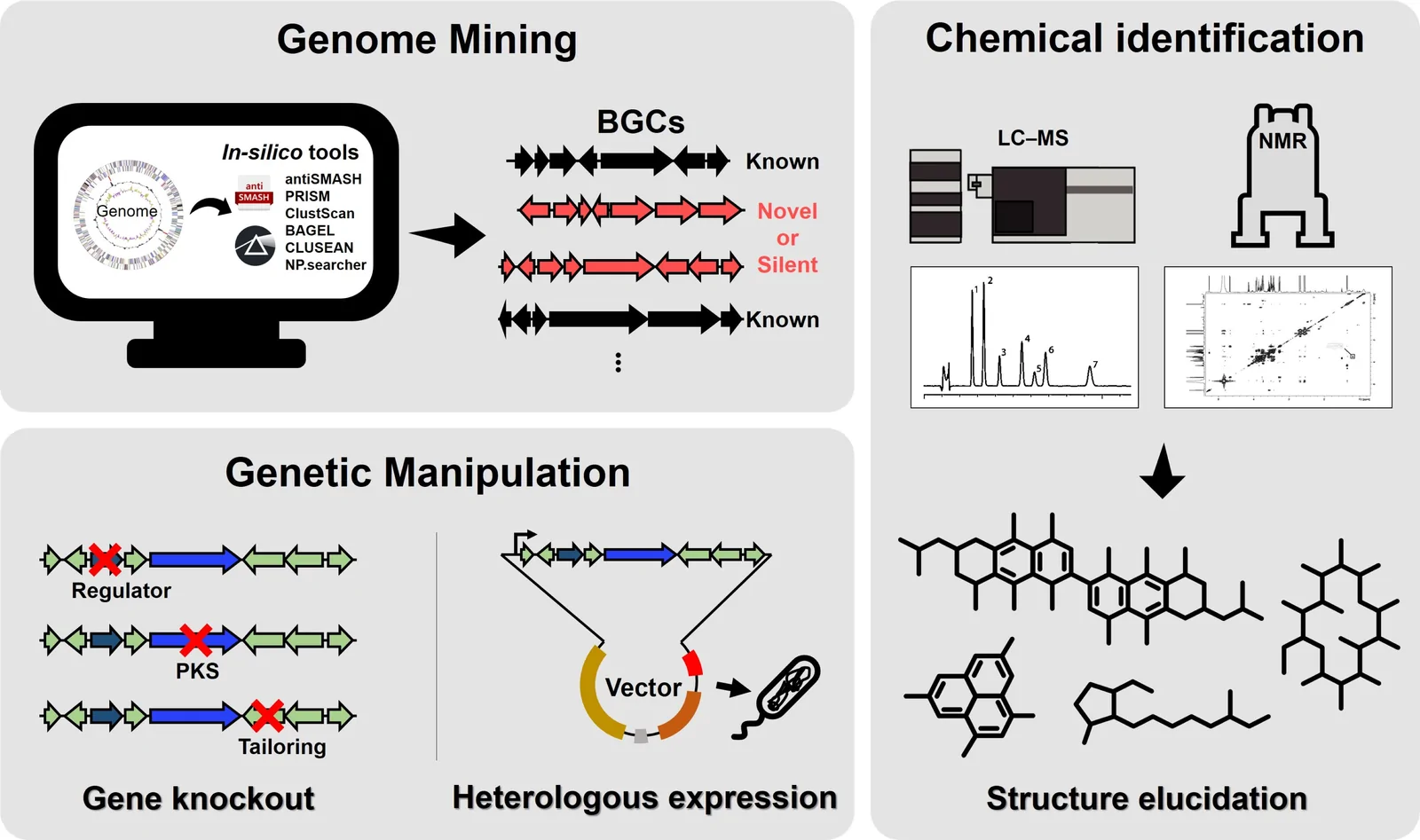
Overall scheme for integrating genome mining, genetic manipulation, and chemical identification aimed at the discovery of natural products. Genome mining employs in silico tools to identify biosynthetic gene clusters (BGCs), which are categorized as known, novel, or silent. Chemical identification utilizes LC-MS and NMR to analyze the metabolites associated with these BGCs and elucidate their chemical structures. Genetic manipulation involves gene knockout to investigate the roles of regulatory or tailoring genes and heterologous expression to validate the production of target compounds. This integrated approach facilitates the discovery and characterization of novel natural products.

The completed genome sequences of *Streptomyces coelicolor* and *S. avermitilis* in 2002–2003 revealed that these organisms encode approximately ten times more secondary metabolites than those identified using traditional microbial cultivation methods (Bentley et al., 2002; Challis, 2014; Ikeda et al., 2003, 2014). These results have also been observed in various *Streptomyces* species and other actinomycete genera (Baltz, 2017; Harrison and Studholme, 2014; Lee et al., 2020). Despite this remarkable genetic capacity, only 3% of the natural products encoded by these genomes have been experimentally characterized as distinct bioactive compounds (Gavriilidou et al., 2022). The slow rate of discovery of new active natural products is not due to the depletion of microbial biosynthetic resources but rather to technical limitations in microbial isolation, cultivation, and bioactivity screening methods. Traditional approaches often fail to induce the expression of biosynthetic gene clusters (BGCs) under laboratory conditions, leaving most secondary metabolites unobserved (Handelsman et al., 1998; Shore and Coukell, 2016). While genome mining has become a crucial strategy for predicting and identifying biosynthetic genes responsible for natural product synthesis by leveraging comprehensive genomic data (Belknap et al., 2020), it does not directly solve the challenge of activating cryptic gene clusters in laboratory conditions. To address this issue, genome mining can be significantly advanced by integrating structural predictions based on bioinformatics, activation of cryptic gene clusters via genetic manipulation, and engineering of natural product analogs (Fig. 1). This integrated approach, which combines gene cluster sequencing with biosynthetic pathway analyses, provides critical advantages for identifying novel compounds. Moreover, it complements efforts to overcome the challenges of inducing gene expression under laboratory conditions, ultimately facilitating the discovery of new bioactive natural products.

(1) Structural prediction and redundancy reduction: Predicting the structures of natural products based on gene sequences allows researchers to identify novel compounds by excluding those with known structures. This targeted approach enhances the probability of identifying unknown natural products by focusing on previously uncharacterized biosynthetic pathways.

(2) Transcriptomic analysis and genetic manipulation: Combining transcriptomic analysis with genetic manipulation of regulatory genes can play a crucial role in the discovery of novel compounds. These methods can help uncover previously silent or cryptic biosynthetic pathways, stimulate the expression of dormant gene clusters, and reveal new metabolites that might otherwise remain undetected. By modifying the expression of genes involved in the biosynthesis of natural products, researchers can activate biosynthetic gene clusters that were previously inactive or under-expressed, thereby expanding the spectrum of natural products available for discovery. This approach not only enhances the identification of novel compounds but also facilitates the characterization of previously unrecognized metabolites with potential bioactivity.

(3) Correlation of structures with biosynthetic pathways: Direct correlation of the chemical structures of natural products with their biosynthetic pathways facilitates the exploration of structural diversity. Researchers can engineer new analogs by studying the mechanisms of biosynthesis and employing combinatorial biosynthesis strategies, further expanding the range of natural products and their potential applications.

These advancements represent a paradigm shift in natural product discovery, offering the potential to exponentially increase the identification of new compounds and broaden our understanding of their biological activities and applications (Ziemert et al., 2016).

Nature often relies on a limited number of mechanisms to produce similar building blocks. This convergence has enabled researchers to uncover new biosynthetic pathways by exploiting the genetic characteristics of the enzymes involved. This protocol provides a comprehensive overview of efficient genome mining strategies for identifying and characterizing specific BGCs and linking them to their corresponding natural products or compound classes. To investigate the relationship between BGCs and their corresponding secondary metabolites, we conducted targeted gene deletion experiments on BGCs identified through antiSMASH predictions. Among these, the SGR-PTM BGC was selected as the primary engineering target due to its distinctive characteristics. Although the structure of SGR-PTM has been previously elucidated, its production has never been detected in the native microbial strain and has only been successfully achieved through heterologous expression. Moreover, the structural features of SGR-PTM strongly suggest its potential for potent antimicrobial activity, highlighting its considerable research value and the urgent need for further investigation. Beyond SGR-PTM, other deletion targets can be selected based on specific criteria. These include putative regulatory genes within the BGCs, as they play a critical role in controlling the expression of biosynthetic pathways. Additionally, structural genes encoding key enzymes involved in the biosynthesis of predicted metabolites should be prioritized, particularly when their roles remain unclear or are hypothesized to significantly influence metabolite production. These criteria provide a systematic and robust framework for identifying and characterizing BGCs with high potential for yielding novel bioactive compounds.

The targeted deletions could be achieved using homologous recombination as guided by this protocol, and their potential effects could be analyzed through comprehensive phenotypic and metabolomic assays. These assays enabled the identification of metabolite production changes and provided insight into the functional roles of the deleted genes within the BGCs. Furthermore, additional genetic manipulations, such as insertions and modifications, could be employed to enhance or modify product yields and to confirm the involvement of specific genes in metabolite biosynthesis. By integrating these targeted approaches, the genetic basis of natural product biosynthesis can be effectively mapped, facilitating the discovery of novel bioactive compounds and expanding our understanding of their potential applications.

## Applications

Genome mining facilitates the detection of biosynthetic pathways for biologically active natural products and allows the prediction of their functions and chemical reactions (Ziemert et al., 2016). This process can be broadly divided into four main strategies: gene cluster identification, structural prediction, cryptic cluster activation, and functional validation. Gene cluster identification utilizes extensive publicly available databases containing genome sequences and annotations. These databases can be combined with computational techniques, bioinformatics algorithms, and tools to identify relevant gene clusters. For instance, several free databases and tools such as BAGEL, ClustScan, CLUSEAN, and NP.searcher have been developed to predict the involvement of BGCs in secondary metabolite production (de Jong et al., 2006; Li et al. 2009; Starcevic et al., 2008; Weber et al. 2009). These tools assist in identifying highly conserved sequences within the genome and map their locations to detect BGCs. However, many of these methods are limited in their ability to detect secondary metabolites, focusing primarily on specific classes such as polyketides and non-ribosomal peptides. In contrast, antiSMASH (Antibiotics and Secondary Metabolite Analysis SHell) and PRISM (PRediction Informatics for Secondary Metabolomes) are the most widely used genome mining tools for predicting various BGC types (Blin et al., 2023; Skinnider et al., 2020). They stand out from other tools because they utilize sequence alignment-based profiles within a Hidden Markov Model (HMM), which is representative of specific BGC families (Weber and Kim, 2016).

antiSMASH, first released in 2011, was designed to identify BGCs across various classes, including polyketides, non-ribosomally synthesized peptides, terpenes, aminoglycosides, aminocoumarins, indolocarbazoles, lantibiotics, bacteriocins, nucleosides, beta-lactams, butyrolactones, siderophores, and melanins. This web server integrates gene cluster identification with a series of specific algorithms for natural compound analysis, offering detailed gene cluster predictions and generating predicted images of amino acid structures. The procedure begins with the identification of homologous genes within the target genome. Predefined cluster rules were then applied to define potential individual clusters encoded in each genomic region based on their similarity to known BGC families. These potential clusters were extended by adding upstream and downstream genes according to the family specific expansion rules to ensure that the predicted BGCs were as comprehensive as possible. This process involved evaluating results using a set of manually curated BGC cluster rules and eliminating false positives with negative models, such as distinguishing homologous fatty acid synthases from polyketide synthases (PKSs). The antiSMASH output provides a detailed schematic profile of each predicted BGC, including the genes within the BGC, their sequences, the family of BGCs to which it belongs, and the homologous BGCs found in antiSMASH, MIBiG, and other relevant databases. It also predicts the roles of each gene and, for specific biosynthetic classes such as PKSs and non-ribosomal peptide synthetases (NRPSs), the core chemical structures (Blin et al., 2023; Medema et al., 2011).

Once the BGCs responsible for natural product synthesis are identified, the next crucial step is to link these BGCs to the specific chemical structures of the products they produce. This can be accomplished by analyzing the amino acid sequences of the enzymes encoded within the BGCs, which allows the prediction of the chemical reactions they catalyze and, consequently, the structure of the resulting products. For instance, bioinformatics tools such as PRISM, a genome analysis toolkit, and web applications have been used to predict polyketide or non-ribosomal peptide structures based on the presence of specific enzyme domains and their catalytic activities (Skinnider et al., 2015). Recently, PRISM 4 has expanded these capabilities, enabling genome-based predictions of chemical structures for all major classes of bacterial natural antibiotics currently in clinical use, including aminoglycosides, nucleosides, β-lactams, alkaloids, and lincosamides (Skinnider et al., 2020). This advancement allows for precise structural predictions by linking biosynthetic genes to the enzymatic reactions they catalyze, enabling the reconstruction of complete biosynthetic pathways and the prediction of final products in silico. This enhanced capability significantly improves the ability to predict and manipulate natural product biosynthesis at the genomic level.

Many secondary metabolite gene clusters in the actinomycete genome remain transcriptionally silent under standard laboratory conditions, meaning that they are not actively expressed and do not produce their associated metabolites without specific environmental triggers or regulatory signals (Craney et al., 2013). Activation of these clusters could lead to the discovery of a vast array of new enzymes, biological pathways, and natural product resources. Several methods can be used to activate cryptic gene clusters. For example, optimizing the cultivation conditions, such as the composition of the culture medium, aeration, fermenter design, lighting conditions, and temperature, can trigger the expression of otherwise dormant genes. Chemical approaches, including the addition of enzyme inhibitors, inducers, or rare-earth elements, can also alter the regulatory networks that control these clusters (Bode et al., 2002; Mohammadipanah et al., 2020; Ochi et al., 2014; Yoon and Nodwell, 2014). Co-culture, in which actinomycetes grow alongside other microorganisms, fosters interspecies interactions that may activate silent gene clusters (Lu and Shen, 2004). More directly, manipulating pathway-specific regulation, such as overexpressing regulatory genes or introducing novel promoters, can activate cryptic gene clusters (Bentley et al., 2002; Li et al., 2018; Ramos et al., 2005). Additionally, by transferring the BGC of interest from its native organism to a more genetically tractable host (model organism) through heterologous expression, researchers can bypass challenges such as slow growth, complex media requirements, and genetic intractability associated with the native producer (Bian et al., 2017; Wang et al., 2018; Zhang et al., 2017).

Finally, functional validation within a microorganism is essential and can be achieved through the targeted modification or manipulation of specific genes. By regulating gene expression either through the inactivation or overexpression of particular genes, phenotypic changes that elucidate the roles of these genes in cellular and organismal functions can be observed. Furthermore, gene manipulation techniques can be integrated with synthetic biology strategies to create novel metabolic pathways by manipulating, expressing, or rearranging multiple biosynthetic genes (Lee et al., 2021). This approach enables the production of unique chemical analogs or secondary metabolites that exceed the capabilities of native producers, ultimately facilitating the discovery of new biologically active compounds or therapeutic agents.

## Methods

### Genome mining by antiSMASH

The antiSMASH framework enables the detection of BGCs that encode secondary metabolites in genomes and offers detailed sequence analysis. It supports various data formats, including Genbank (recommended), EMBL, and plain FASTA. Depending on the type of organism, the following websites can be used: https://antismash.secondarymetabolites.org for bacterial sequences, https://fungismash.secondarymetabolites.org for fungal sequences, and http://plantismash.secondarymetabolites.org for plant sequences.

When analyzing draft genome sequences, it is advisable to use scaffold sequences that include 'N' characters in the gaps, as gene clusters can only be identified when location information is available. The quality of the input data is critical, as fragmented or low-quality sequences can hinder the accurate prediction of gene clusters. High-quality genome assemblies significantly improve the reliability of the results.

The analysis process begins with preparing genomic data in an appropriate format, with Genbank being the most suitable due to its detailed annotations. Once the genome data is ready, it is uploaded to the appropriate antiSMASH platform, depending on the organism of interest. Users can customize the analysis by selecting organism-specific parameters and enabling advanced features, such as domain detection and subcluster annotation, which provide more comprehensive insights into the biosynthetic potential of the genome.

The antiSMASH framework scans the uploaded genome to detect BGCs, classify their types, and generate detailed reports. These reports include annotated genomic maps and predictions about the metabolites potentially produced by each detected cluster. The results are downloadable for further analysis. To ensure the accuracy of predictions, it is advisable to validate the output by reviewing the detected clusters and verifying their completeness. False positives can occur, especially with incomplete draft genomes, so cross-referencing results with other bioinformatics tools or manual curation is recommended.

By leveraging high-quality genome data and applying antiSMASH alongside rigorous validation steps, researchers can systematically identify and characterize biosynthetic gene clusters. This approach is instrumental in discovering new bioactive compounds and expanding our understanding of natural product biosynthesis.

### Genetic manipulation system for *Streptomyces* using conjugal transfer

The genetic manipulation of *Streptomyces* species is crucial for studying gene function, producing secondary metabolites, and engineering strains for biotechnological applications. Several transformation techniques have been developed, particularly in the model strain S. coelicolor A3(2) (Kieser et al., 2000). One commonly used method is protoplast transformation, which facilitates the transfer of foreign DNA into *Streptomyces*. However, the conditions required for successful protoplast formation and transformation can vary significantly between different *Streptomyces* species and strains, making it difficult to standardize protocols (Baltz, 1998).

In 1983, the demonstration of intergeneric plasmid transfer from *Escherichia coli* to *Streptomyces* strains led to the development of an alternative method for transferring constructs (cloned plasmids/cosmids) into *Streptomyces* (Matsushima et al., 1994; Simon et al., 1983). The conjugation of *E. coli* plasmid DNA is mediated by the *oriT* function derived from plasmid RK2, facilitating its integration into the *Streptomyces* chromosome through homologous recombination. This procedure has been applied to various actinomycetes and has demonstrated high efficiency compared to gene transfer achieved through protoplast transformation (Blaesing et al., 2005; Mazodier et al., 1989).

## Materials

### Reagents

• LB broth: 10 g/L trypton, 5 g/L yeast extract, 10 g/L NaCl

• 2X YT broth: 16 g/L trypton, 10 g/L yeast extract, 10 g/L NaCl

• MS medium: 20 g/L mannitol, 20 g/L soya flour, 20 g/L agar

• Antibiotics: nalidixic acid (25 mg/ml), chloramphenicol (50 mg/ml), kanamycin (50 mg/ml) and apramycin (50 mg/ml)

• *E. coli* donor strain ET12567/pUZ8002 containing conjugative plasmid

• Acceptor *Streptomyces* strain

### Equipment

• Heating block (Lab-Line)

• Incubator (BioFree)

• Falcon centrifuge (LABOGENE)

• Micro-centrifuge (LABOGENE)

• Vortexer (Scientific Industries)

### Others

• antiSMASH bacterial version tool (antiSMASH 7.1)

• Whole genome sequences of *Streptomyces*

### Protocols

This protocol is designed to identify the BGCs of secondary metabolites from the whole genome sequences of *Streptomyces* species using the antiSMASH tool. It also describes methods for manipulating target biosynthetic genes within these organisms to verify BGCs and validate their functions. Detailed cloning methods, including DNA preparation, enzyme digestion, PCR, transformation, and sequencing, are not included in this protocol, as they are well-optimized in each laboratory. All procedures should be conducted at room temperature unless otherwise specified.

#### A. Prediction of secondary metabolite BGCs in the genome of Streptomyces using antiSMASH

1. Upload a FASTA file or enter the gene accession number into the data input box.

Note: GenBank files are uploaded to the antiSMASH server using the default parameters. It is recommended to use the gene annotation file (GBK/EMBL) obtained from Rapid Annotations via Subsystems Technology (RAST) (Aziz et al., 2008). However, if gene annotations are not available, a simple FASTA file can also be used.

• Note: While entering your email address is optional, providing it is recommended so that you can receive an email notification once the results have been processed.

• Note: The 'Extra Features' panel enables the addition of supplementary databases or repositories for searching to assist in identifying clusters. For general bacterial sequence analysis, the standard antiSMASH parameters work well with five core features: KnownClusterBlast, SubClusterBlast, ActiveSite-Finder, RREFinder, and TFBS analysis. Additionally, the antiSMASH run can be extended by enabling five more features: ClusterBlast, MIBiG cluster comparison, Cluster Pfam analysis, Pfam-based GO term annotation, and TIGRFam analysis.

When the 'Submit' button is clicked, the analysis and identification of clusters are performed on the server. Once the task is complete, the results will be sent to the email address provided at submission.

**Fig. 2.**
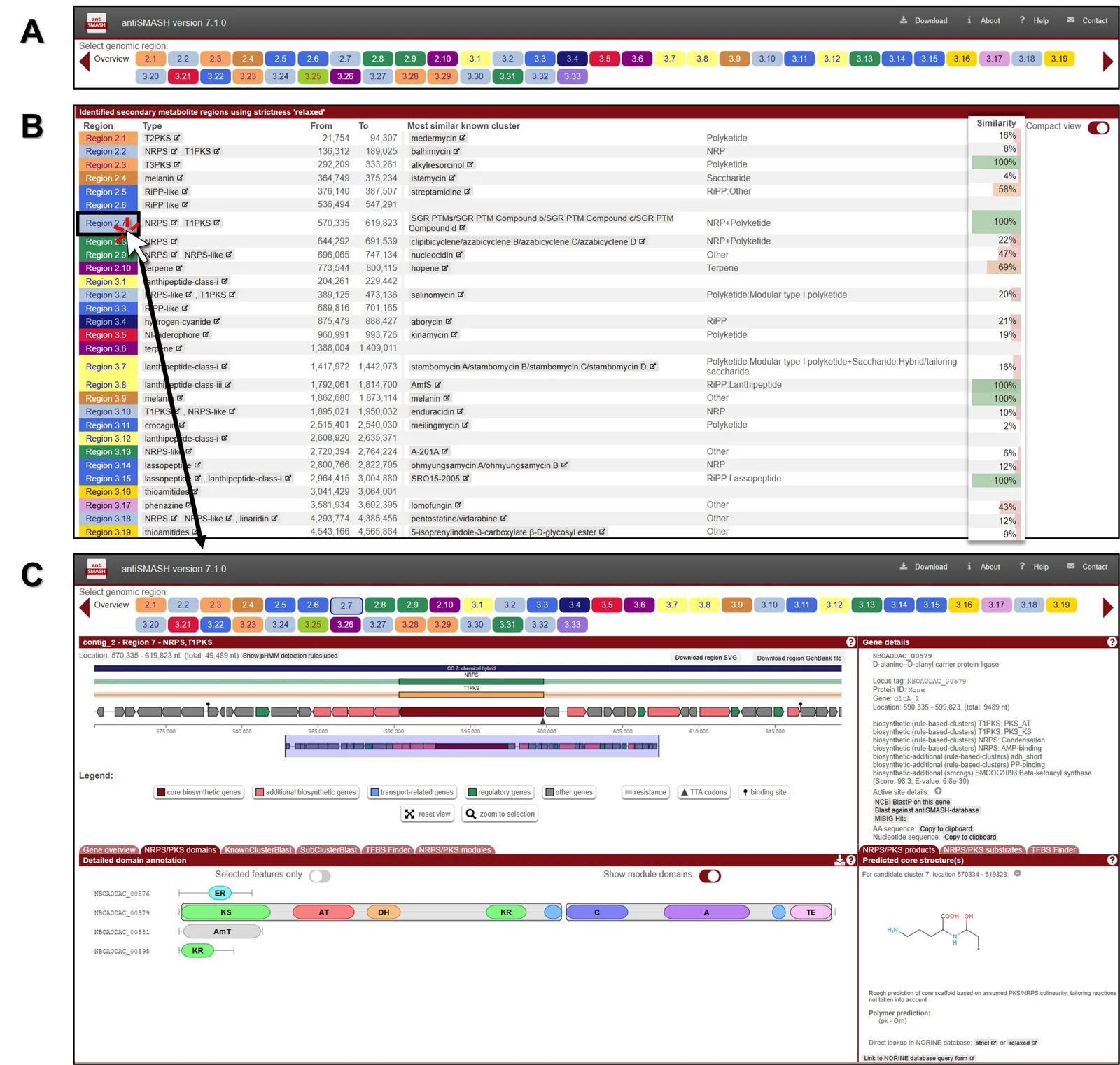
Screenshot of genome mining and annotation analysis of a novel *Streptomyces* strain using bacterial antiSMASH 7.1. (A) Overview table of BGCs. (B) Identified secondary metabolite regions. (C) Detailed analysis results for each individual BGC. A detailed description is provided in the protocol.

2. Note: For a typical bacterial genome, the process takes approximately 0.5 to 2 h under normal server load.

3. The analysis identified 48 secondary metabolite regions in our query, and the colored circles at the top of the screen represent each identified gene cluster (Fig. 2A). Below is a detailed list of the clusters analyzed for *Streptomyces*, including the coordinates of the identified gene clusters (Fig. 2B).

4. To analyze the corresponding BGC for the identified group, click on one of the cluster leads represented by the colored circles. For this analysis, the Region 53.1 PKS-NRPS hybrid cluster has been selected (Fig. 2C).

• Note: Clicking on an individual gene arrow in the annotated BGC retrieves detailed characteristics and features of that gene.

• Note: Generally, each type of PKS module consists of several domains, each separated by short spacer regions and performing specific functions. The description below primarily focuses on type I PKS, which is a modular, multi-domain enzyme system. The order of modules and domains in a complete type I PKS, from N-terminal to C-terminal, is as follows: the initiation or loading module includes the acyltransferase (AT) and acyl carrier protein (ACP); the elongation or extension module consists of the ketosynthase (KS) and AT, and may also include the dehydratase (DH), enoyl reductase (ER), and ketoreductase (KR), all leading to the ACP; and the termination or release domain is represented by the thioesterase (TE).

• Note: The order of modules and domains in a complete NRPS is as follows: it begins with the initiation or loading module, which consists of the adenylation (A) domain and the peptidyl carrier protein (PCP). This is followed by the elongation or extension module, which includes the condensation (C) domain, an additional A domain, and another PCP. Finally, the process concludes with the termination or release module, denoted as the thioesterase (TE) domain. This sequence progresses from the N-terminal to the C-terminal.

• Note: KnownClusterBlast displays comparisons with known (and characterized) BGCs from the MIBiG dataset. Subcluster Blast analysis identifies similarities between operons within the BGCs. TFBS Finder predicts transcription factor binding sites using LogoMotif profiles.

• Note: On the right side of the window, the core structure inferred from the biosynthetic enzymes is displayed, along with the predicted details. However, this is only a rough prediction of the core scaffold based on assumed PKS/NRPS collinearity, and it does not account for any tailoring modifications or non-standard reactions.

5. The download button at the top of the antiSMASH results page allows users to download all or some of the results. Analysis results are stored on the server for one month before being deleted.

#### B. Establishment of a method for in-frame gene deletion using the conjugal transfer system in Streptomyces sp.

##### B-1. Preparation of donor *E. coli* cell

**Fig. 3.**
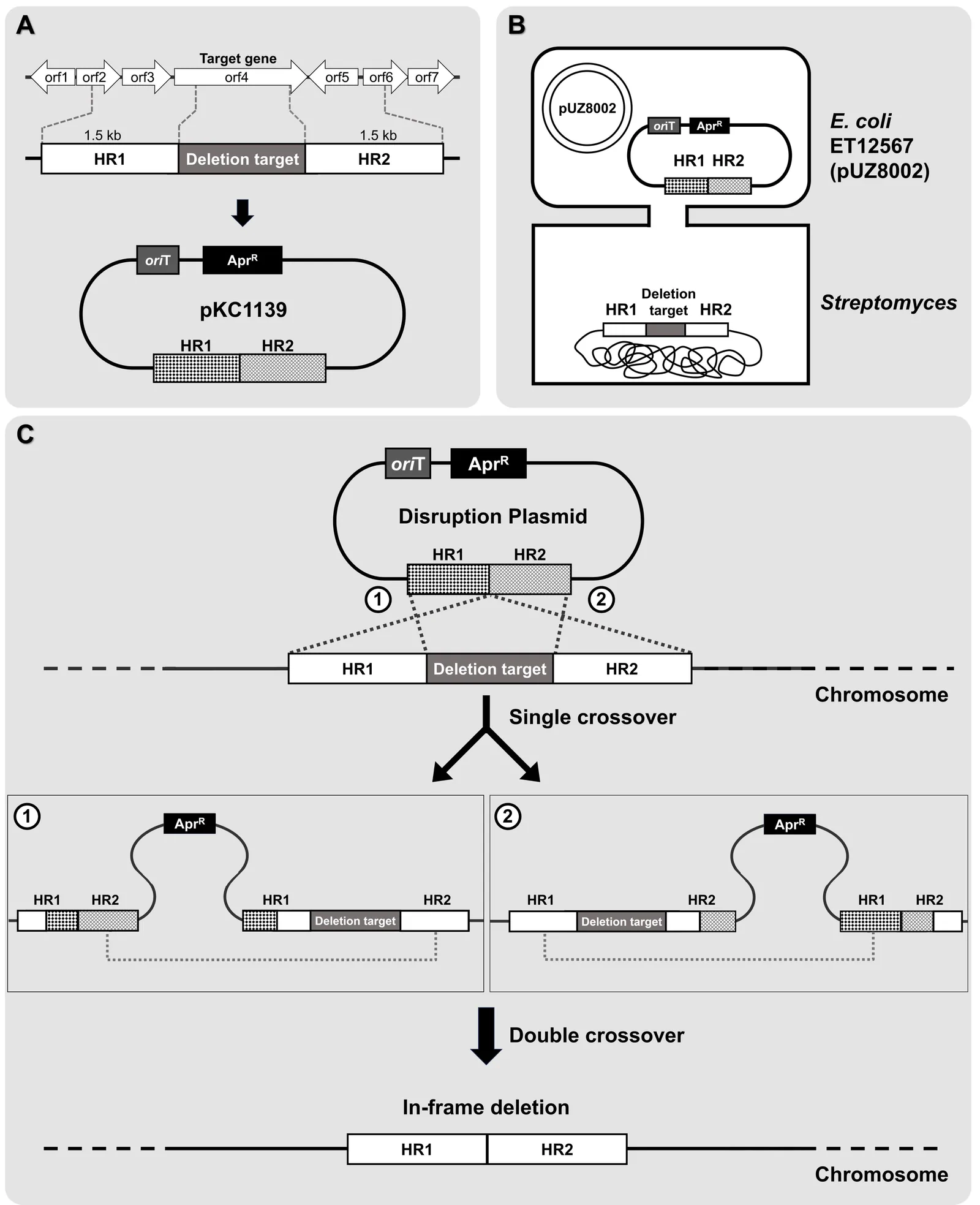
Schematic of in-frame deletion mutant generation via double crossover. (A) Design and construction of the conjugative plasmid. (B) Intergeneric conjugation between *E. coli* and *Streptomyces* species. (C) Double-crossover homologous recombination. A detailed description is provided in the protocol.

1. *E. coli* ET12567 (pUZ8002) containing the conjugative plasmid was pre-grown overnight in LB medium.

• Note: Gene in-frame deletion refers to the removal of a portion of a gene without disrupting the reading frame of the remaining sequence. This ensures that the deleted segment does not introduce frameshifts, maintaining the integrity and functionality of the downstream sequence, which is crucial for studying the function of the target gene. In the context of identifying novel BGCs, this approach is used to systematically inactivate or delete specific genes within the cluster, such as regulators, enzymes, or other components, in order to assess their role in the biosynthetic pathway. By deleting a potential regulatory gene, we can determine its influence on the expression of the biosynthetic cluster, revealing novel regulatory mechanisms or pathways involved in the production of bioactive metabolites. Additionally, this method can be applied to delete entire clusters or individual genes within a cluster to compare metabolite production with that of the progenitor cells, which can provide insight into the functional role of the gene cluster and its associated metabolites. To achieve this, approximately 1.5 kb DNA fragments containing the upstream and downstream sequences of the target gene were designed to function as homologous regions (denoted as HR), amplified by PCR, and subsequently cloned to construct the conjugative plasmid (Fig. 3A). Clarifying the specific objective of this in-frame deletion strategy, whether it is to investigate gene regulation or compare metabolite production, is crucial for understanding its relevance in identifying and characterizing novel BGCs.

• Note: pKC1139 plasmid contains an apramycin resistance gene for selection, an *oriT* sequence for conjugal transfer, and origins of replication for both *E. coli* and *Streptomyces*. The *Streptomyces* origin of replication, derived from *S. ghanaensis*, functions only at temperatures below 34°C (Muth et al., 1989). Therefore, under selective pressure at non-permissive temperatures, the plasmid must integrate into the chromosome through homologous recombination to be stably maintained. To construct the pKC1139-derived deletion vectors used in the proof-of-concept experiment, we employed a Gibson assembly method. This approach allowed for the seamless integration of DNA fragments, including the upstream and downstream flanking regions of the target gene, into the pKC1139 backbone. While specific reaction conditions are not detailed here, this method ensured the precise assembly of the deletion vectors for use in conjugation and subsequent homologous recombination experiments.

• Note: The conjugative plasmid must be transformed into the *E. coli* strain ET12567 (pUZ8002), which serves as a convenient donor due to its methylation deficiency that allows it to circumvent the methylation-specific restriction mechanisms employed by some *Streptomyces* species to protect against foreign DNA.

2. 1 ml of the overnight culture was inoculated into 50 ml of the same medium in a 250 ml Erlenmeyer flask and incubated at 37°C with shaking at 200 rpm until the OD_600_ reached 0.4–0.6.

3. The cultures were harvested by centrifugation and washed three times with an equal volume of antibiotic-free LB medium.

4. The harvested *E. coli* cells were resuspended in 500 μl of 2X YT medium.

##### B-2. Preparation of receptor *Streptomyces* spore

1. The spores of *Streptomyces* were washed twice and suspended in 2X YT medium at a concentration of 10^9^ spores/ml.

2. The spores were heated at 45–60°C for 10 min to synchronize germination, and then allowed to cool down to room temperature (RT).

• Note: To prepare the spores, spread the desired *Streptomyces* strain on MS agar medium, typically incubated at 28–30°C for several days until sporulation is observed. Sporulation can be recognized by a grayish coloration, as the spores are pigmented. The spores were then resuspended in 1 ml of 20% glycerol and preserved in 50 μl aliquots at -80°C (Segura et al., 1996).

##### B-3. Intergeneric conjugation and overlay

1. Donor cells (500 μl *E. coli*) were added to recipient cells (500 μl of heat-shocked spores).

2. The mixture was gently mixed, centrifuged at 13,000 rpm for 1 min and the supernatant removed.

3. The resulting cell pellet was resuspended in 100 μl of 2X YT medium. Dilutions of the cell mixture (10^-1^ to 10^-3^) were prepared using a maximum volume of 200 μl of 2X YT, which was then spread onto Petri dishes containing 30 ml of MS medium supplemented with 10 mM MgCl_2_ (without antibiotics) and incubated at 30°C for 16 to 20 h.

• Note: The concentration of MgCl_2_ (10–40 mM) can be adjusted according to the conjugal efficiency.

4. After overnight incubation, the conjugation plates should be overlaid with nalidixic acid (0.5 mg) and plasmid selection antibiotics (1–4 mg) to select the appropriate exconjugants. Incubation is then continued at 30°C until exconjugant cells appear.

• Note: A 500 µl solution for each plate should be prepared using sterile distilled water and the required antibiotic concentration, ensuring thorough mixing. A sterilized 'L'-shaped spreader can be used to evenly distribute the solution across the entire surface of the plate while minimizing contact.

• Note: As shown in Fig. 3A, the plasmid used in this study confers resistance to apramycin, which means that all successful exconjugants will exhibit this resistance. Furthermore, when applied at the recommended concentrations, nalidixic acid effectively inhibits the growth of *E. coli*.

5. Potential exconjugants are plated on media containing nalidixic acid and apramycin to select for resistant colonies. After selection, single colonies are picked and streaked onto fresh media with the same antibiotics, then incubated at 37°C. This incubation step eliminates any remaining *E. coli* cells and promotes integration of the plasmid into the chromosome through single crossover events (Fig. 3C).

• Note: If a single crossover event occurs, the entire plasmid integrates into the genome, resulting in clones that carry the apramycin resistance gene. The integration of the plasmid is confirmed by PCR, using primers designed to amplify the specific genomic region where the plasmid has been inserted.

6. Single crossover colonies are then cultured repeatedly in antibiotic-free media to promote double crossover recombination events. To verify these events, several single colonies should be selected and replica-streaked onto MS agar supplemented with apramycin and onto MS agar without antibiotics for maintenance. If the colonies fail to grow under apramycin selection, this confirms the occurrence of the double crossover recombination event.

7. To determine if recombination has occurred at the desired location, PCR tests are performed using two primers that anneal outside the boundaries of the recombination site. The length of the PCR product should differ from that of the wild-type strain. As the PCR amplification length serves only as a preliminary indicator, further confirmation is achieved through Sanger sequencing (Fig. 3C).

8. Transgenic colonies with confirmed genotypes are prepared as spore suspensions for preservation.

## Expected Results

### Rediscovery of SGR-PTM production in a newly isolated *Streptomyces* sp.

**Fig. 4.**
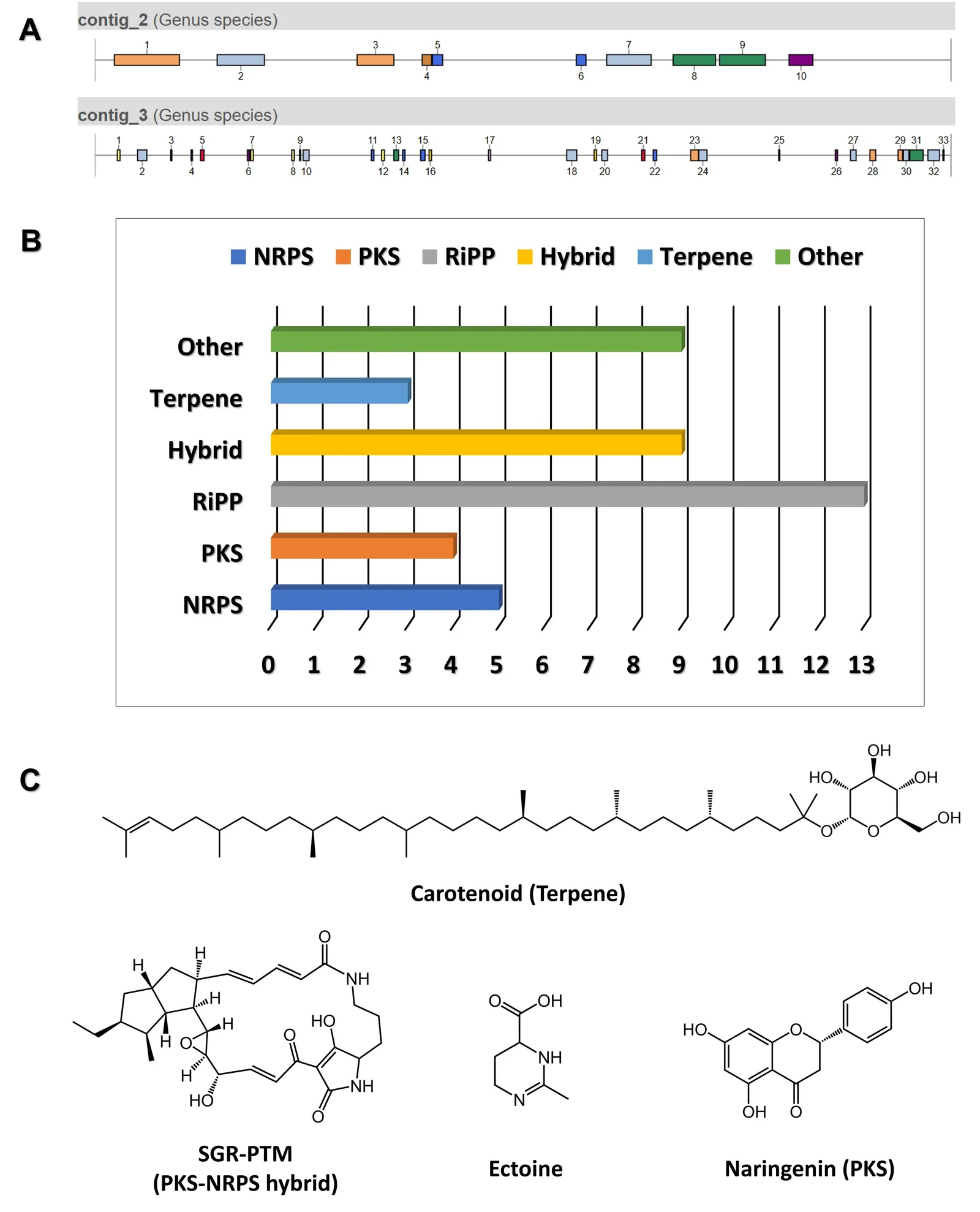
antiSMASH data analysis. (A) Distribution of 43 gene clusters identified across the analyzed genomes. (B) Distribution of various types of gene clusters, categorized by their functional annotations. (C) Examples of predicted compounds identified in the analysis.

The genome of a newly isolated *Streptomyces* sp. from a limestone cave in Gangwon State, South Korea, was sequenced and found to be approximately 8.91 Mb in length (unpublished data). Using antiSMASH version 7.1, 43 gene clusters were predicted from the genome (Fig. 4A). Among these, 10 BGCs showed 100% similarity to known clusters, one exhibited 72% similarity, and the remaining clusters demonstrated less than 70% similarity, indicating the potential for novel or unique natural product biosynthesis. Notably, the predicted BGCs included various categories: ribosomally synthesized and post-translationally modified peptides (Ripps) (13), NRPS (5), PKS (4), NRPS-PKS hybrids (9), terpenes (3), and others (9) (Fig. 4B).

**Fig. 5.**
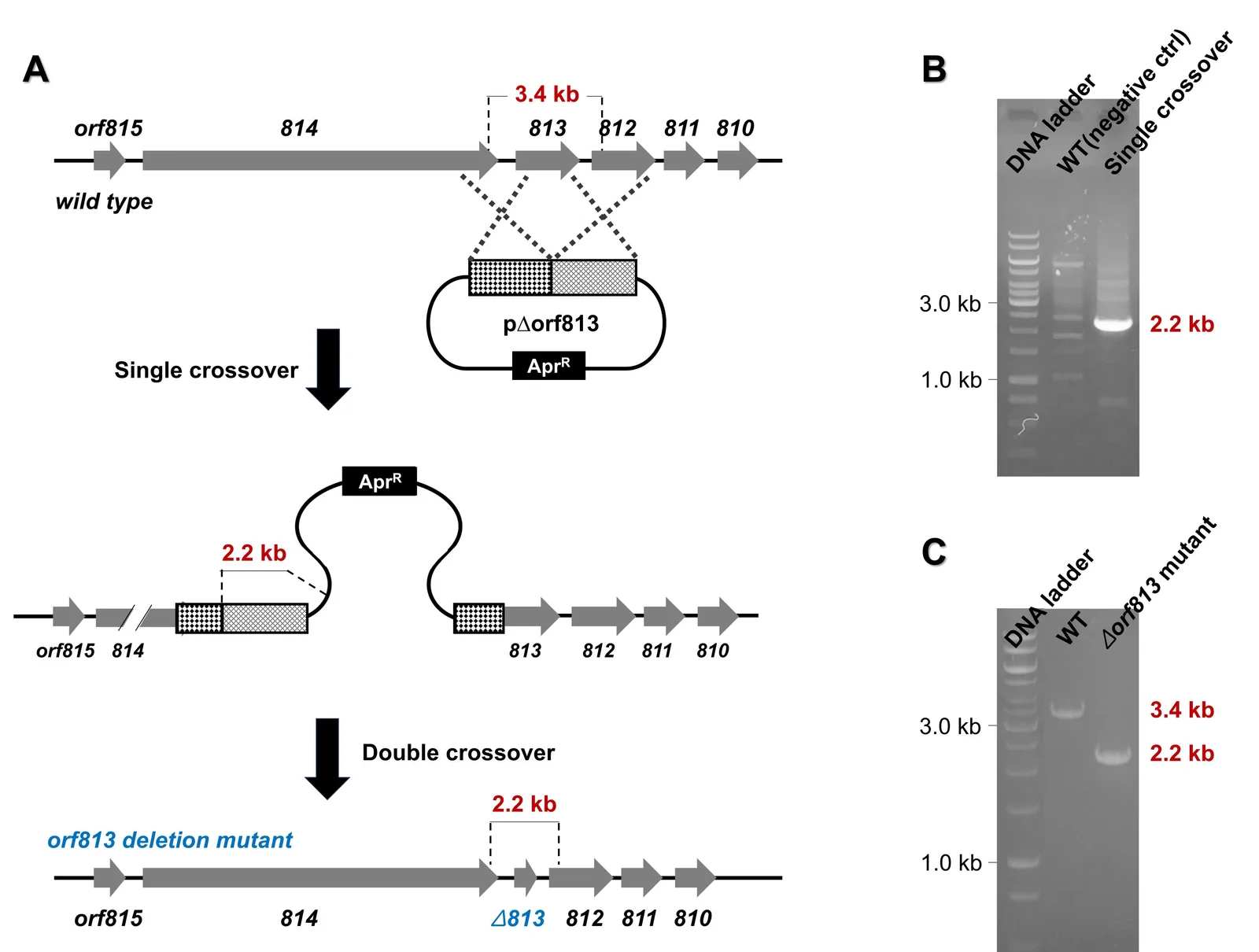
Generation of a novel *Streptomyces* sp.-based mutant with an in-frame deletion of the *orf813* gene (∆orf813). (A) Schematic representation of the in-frame deletion of *orf813* using the p∆orf813 construct. (B) PCR analysis confirming the occurrence of the single crossover event. (C) PCR analysis verifying the generation of the *orf813* gene deletion mutant (∆orf813).

Examples of compounds predicted to be produced by conserved BGCs include those shown in Fig. 4C, such as SGR-PTM. SGR-PTM is characterized by the presence of an epoxide ring, which is hypothesized to contribute significantly to their bioactivity. Although their biosynthetic pathways have been elucidated through heterologous expression (Luo et al., 2013; Ohnishi et al., 2008), no specific biological activity has been identified to date. Furthermore, their productivity in the native producer strain is extremely low, presenting significant challenges for further investigation. Despite these limitations, we successfully applied the methods outlined in this protocol to identify a natural producer strain of SGR-PTM. To validate the role of the predicted BGC in SGR-PTM biosynthesis, we performed a targeted deletion of an essential biosynthetic gene within the cluster (Fig. 5). The deletion mutant was unable to produce SGR-PTM, conclusively establishing the involvement of this BGC in SGR-PTM biosynthesis (unpublished data). Overcoming the low productivity of SGR-PTM in its native producer strain is essential for advancing both research and potential applications. Promising genetic engineering approaches include replacing weak native promoters with stronger ones or enhancing the expression of rate-limiting enzymes within the biosynthetic pathway. These strategies not only have the potential to significantly boost SGR-PTM production but also enable the creation of structurally diverse derivatives, thereby expanding its range of bioactivity.

### Genome-based discovery strategies for novel natural products

Genome-based discovery tools, such as antiSMASH, are highly accurate in predicting biosynthetic gene clusters (BGCs) and their potential to produce natural products. However, while these tools can identify candidate BGCs, they cannot inherently determine whether the encoded products exhibit biological activity. This limitation can be addressed by focusing on BGCs associated with biosynthetic pathways that produce compounds containing biologically active functional groups, such as epoxide rings, enediynes, β-lactones, and epoxyketones. As demonstrated in this protocol, targeting these biologically active features can effectively guide genome mining strategies. These functional groups often play a crucial role in covalent interactions with protein targets, thereby driving the bioactivity of the compounds (Eustaquio et al., 2011; Owen et al., 2015; Yan et al., 2016, 2017).

Identifying the enzymes responsible for incorporating such functional groups into natural products can further refine genome mining strategies. For example, focusing genome mining efforts on orphan BGCs encoding these functional motifs has the potential to uncover new bioactive compounds. Additionally, characterizing the biosynthetic pathways responsible for these features provides valuable insights into their underlying mechanisms, enabling the rational design of analogs with enhanced or novel properties. By integrating genome mining with advanced biosynthetic and chemical analyses, this approach offers a promising pathway for the discovery of innovative natural products with significant biological potential.
